# Catheter ablation for atrial fibrillation in patients with persistent left superior vena cava: Case series and systematic review

**DOI:** 10.3389/fcvm.2022.1015540

**Published:** 2022-10-17

**Authors:** Mingyang Gao, Yang Bian, Lihong Huang, Jingrui Zhang, Changyi Li, Nian Liu, Xiaoxia Liu, Song Zuo, Xueyuan Guo, Wei Wang, Xin Zhao, Deyong Long, Caihua Sang, Ribo Tang, Songnan Li, Jianzeng Dong, Changsheng Ma

**Affiliations:** ^1^Department of Cardiology, Beijing Anzhen Hospital, Capital Medical University, Beijing, China; ^2^Department of Cardiology, Baoji Hospital Affiliated to Xi’an Medical University, Baoji, Shaanxi, China

**Keywords:** catheter ablation, atrial fibrillation, persistent left superior vena cava, radiofrequency ablation, cryoballoon

## Abstract

**Introduction:**

Persistent left superior vena cava (PLSVC) is the most common form of thoracic venous abnormality. Catheter ablation (CA) for atrial fibrillation (AF) can be complicated by the existence of PLSVC, which could act as an important arrhythmogenic mechanism in AF.

**Methods and results:**

We reported a case series of patients with PLSVC who underwent CA for AF at our center between 2018 and 2021. A systematic search was also performed on PubMed, EMBASE, and Web of Science for research reporting CA for AF in patients with PLSVC. Sixteen patients with PLSVC were identified at our center. Ablation targeting PLSVC was performed in 5 patients in the index procedures and in four patients receiving redo procedures. One patient experienced acute procedure failure. After a median follow-up period of 15 months, only 6 (37.5%) patients remained free from AF/atrial tachycardia (AT) after a single procedure. In the systematic review, 11 studies with 167 patients were identified. Based on the included studies, the estimated prevalence of PLSVC in patients undergoing CA for AF was 0.7%. Ablation targeting PLSVC was performed in 121 (74.7%) patients. Major complications in patients with PLSVC receiving AF ablation procedure included four cases of cardiac tamponades (2%), three cases of cardiac effusion (1.5%), one case of ischemic stroke, and three cases of phrenic nerve injury (1.5%) (one left phrenic nerve and two right phrenic nerve). Pooled analysis revealed that after a median follow-up period of 15.6 months (IQR 12.0–74.0 months), the long-term AF/AT-free rate was 70.6% (95% CI 62.8–78.4%, *I*^2^ = 0.0%) (Central illustration). Different ablation strategies for PLSVC were summarized and discussed in the systematic review.

**Conclusion:**

In patients with PLSVC, recurrence of atrial arrhythmia after CA for AF is relatively common. Ablation aiming for PLSVC isolation is necessitated in most patients. The overall risk of procedural complications was within an acceptable range.

## Introduction

Persistent left superior vena cava (PLSVC) is the most common type of thoracic vein abnormality, with an estimated prevalence of 0.3–0.5% in the general population (1) and 4–8% in patients with congenital heart disease (CHD) (2). It results from the persistent patency of the left cardinal vein which failed to undergo the embryological transformation to the ligament of Marshall. PLSVC can have drainage into a dilated coronary sinus (CS) or directly into the left atrium (LA), constituting a potential cause of right-to-left shunt.

Although often asymptomatic and hemodynamically insignificant, the existence of PLSVC can exert a great impact on interventional procedures, especially for cardiac electrophysiologists, for it not only increases the complexity of vascular access (3) but also serves as a potential arrhythmogenic origin, *per se*, especially in patients with atrial fibrillation (AF). Previous investigations demonstrated that PLSVC plays an important role in both the initiation and maintenance of AF, and targeted ablation within PLSVC has been reported in several cases (4–6).

Due to its relatively low prevalence, a universally accepted catheter ablation (CA) strategy for AF in patients with PLSVC has not been established. We reviewed all patients with PLSVC who received CA for AF at our center. To get an overview of the current practice and evaluated the impact of PLSVC on CA for AF, we also performed a systematic review of the relevant literature.

## Materials and methods

### Single-center case series

#### Ablation procedure and follow-up

All patients who underwent CA for AF at our center between September 2018 and April 2022 were screened for patients with PLSVC. General principles of perioperative management and ablation protocol at our center have previously been described in detail (7, 8). Three-dimensional electroanatomical mapping was performed with multipolar electrodes (PentaRay; Biosense Webster, Diamond Bar, CA, USA) under the guidance of the CARTO 3 system (Biosense Webster, Diamond Bar, CA, USA). Routine ablation strategy at our center included pulmonary vein isolation (PVI) for paroxysmal AF (PAF) and a ‘2C3L’ protocol for persistent AF (PsAF) (7) (PVI and linear ablation at LA roofline, mitral isthmus [MI] line and cavotricuspid isthmus [CTI] line). Complex fractionated atrial electrograms (CFAE) ablation was also allowed at the operators’ discretion. If AF sustained upon the completion of these routine steps, electrical cardioversion would be performed to restore sinus rhythm (SR). Under SR, PVI and linear block would be verified followed by necessary touch-up ablation. At last, burst pacing from the right atrium with a cycle length of 200–300 ms for 10 s would be performed after the completion of PVI and linear blocks. No drug testing by isoproterenol or adenosine is routinely performed. If triggering activities initiating AF, atrial flutter/tachycardia, as well as frequent premature atrial contractions > 10/min appeared spontaneously or induced by burst pacing, we would roughly locate the origin by the activation sequence of the decapolar catheter positioned in CS, followed by a detailed activation mapping to confirm the exact origin. If spontaneous ectopies originating from PLSVC were observed, ablation within PLSVC would be performed aiming for PLSVC isolation, with lesions targeting LA-PLSVC and CS-PLSVC connections. Prominent potentials within PLSVC would also be eliminated.

A standard irrigated-tip ablation catheter (Thermocool SmartTouch SF, Biosense Webster, Diamond Bar, CA, USA) was used in all radiofrequency ablation procedures. PVI was performed with a power of 40–50 W, and linear ablation with 35–40 W. Ablation within PLSVC was performed with a power set at 25–35 W and a saline irrigation rate of 17–30 mL/min, targeting an ablation index of 350–400 at the operator’s discretion. All ablation was conducted in a point-by-point fashion under the power-controlled mode, with a contact force between 10 and 20 g, and an inter-tag distance of ≤6 mm. The endpoint of the ablation procedure was PVI, as well as complete linear block and PLSVC isolation if such interventions were conducted.

All patients were follow-up at 1, 3, 6 months, and every 6 months thereafter. Twelve lead surface ECGs, as well as a 24-h Holter, were requisite for every follow-up visit. This study adheres to the guiding principles of the Helsinki Declaration and is approved by the ethnic institute of Beijing Anzhen Hospital.

#### Statistical analysis

Data were expressed as mean ± standard deviation or median with an interquartile range for continuous variables, and as number (frequency) for categorical variables. Continuous variables were compared using the Student’s *t*-test or Mann–Whitney *U* test, while categorical variables were compared using the chi-square test or Fisher’s exact test. A *P*-value < 0.05 was considered statistically significant. All analyses were conducted using SPSS 26.0 software.

### Systematic review

#### Search strategy and study selection

A systematic search in PubMed, EMBASE, and the Web of Science databases was performed on 2 July 2022 for publications from the year 2,000 onward, utilizing combinations of the relevant medical subject heading (MeSH) terms, keywords, and word variants for ‘left superior vena cava,’ ‘atrial fibrillation,’ and ‘catheter ablation.’ Studies with the following characteristics were considered eligible: (1) reported CA procedures in human participants who have a confirmed diagnosis of PLSVC and AF; (2) provided a minimum information on patients’ demographics and safety or efficacy data of ablation procedure. Supplementary material provide a complete and detailed description of the systematic review process.

#### Study selection and critical appraisal

Two reviewers (MG and YB) independently screened the identified records for eligibility. Disagreements between reviewers were resolved by consultation with a third senior electro-physiologist (SL). An assessment of the risk of bias and methodological quality on the study level was also conducted independently by these two reviewers, with the usage of a modified form of the Newcastle Ottawa Scale (9).

#### Data extraction and statistical analysis

Two investigators (MG and YB) independently conducted data collection. Statistical analyses were performed using Stata (Version 12.0. College Station, TX, USA). Data were pooled using random-effects, according to the Mantel–Haenszel model. The 95% confidence interval (CI) was used. A two-sided *p*-value of < 0.05 was considered statistically significant. Heterogeneity was quantified using the inconsistency index (*I*^2^). If *I*^2^ < 25%, 25–75%, and >75%, the heterogeneity was considered as low, moderate, and high, respectively. Funnel graph and Egger’s tests were performed to examine the risk of publication bias.

## Results

### Single-center case series

#### Baseline and procedural characteristics

From September 2018 to April 2022, 16 patients with PLSVC among 8421 patients who underwent CA for AF at our center were identified (0.19%, mean age 56 ± 14 years old, nine males, nine PAF). One patient has a history of uncorrected atrial septum defect (patient #9) and one has a history of aortic valve replacement (patient #16). Two patients (#6 and #13) received PVI at other institutions and suffered from AF recurrence 3 and 8 months after the index ablation, respectively. In five patients, preprocedural transthoracic echocardiography (TTE) failed to discover the existence of PLSVC, which was only detected by intracardiac echocardiography (ICE).

**FIGURE 1:**
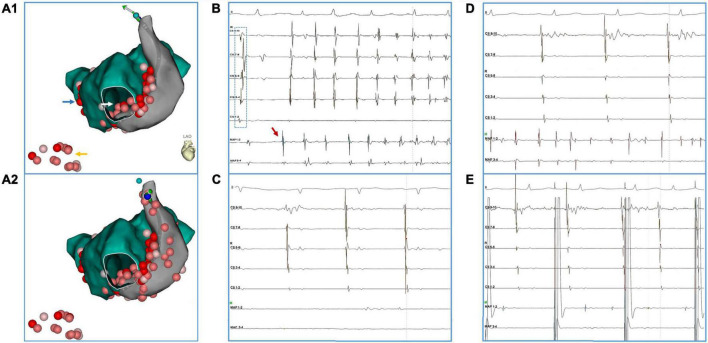
A representative case of PLSVC isolation. The patient (#16) had AF recurrence after a previous ablation procedure in which only PVI was performed. **(A1,A2)** Blue arrow: touch up ablation at RPV. Yellow arrow: linear ablation at CTI. White arrow: CFAE at inferior LA. **(B)** Ectopy (red arrow) from distal PLSVC triggered an episode of AF. Notably, during the sinus beat, activation at the CS catheter presented a bracket-like sequence, which was probably caused by an earlier breakthrough at mid-PLSVC by LA-PLSVC connections. **(C)** After ablation at LA-PLSVC connections [Visitag points in PLSVC in **(A1)**], AF terminated and sinus rhythm was restored. However, a mapping catheter positioned at the distal PLSVC could still record fibrillatory activities. **(D)** Further ablation at distal PLSVC (beyond the level of left superior PV) and ablation at the CS-PLSVC connections was conducted [Visitag points in PLSVC in **(A2)**] which resulted in the elimination of local potential and loss of capture of LA during pacing from distal PLSVC **(E)**. (Abbreviations same as those in the main body).

During the procedure, triggering activities originating from PLSVC were documented in 5 (31%) patients in the index procedure and necessitated ablation in PLSVC. A representative case of PLSVC isolation is presented in Figure 1. No significant difference existed in baseline characteristics between patients who received PLSVC ablation and not received PLSVC ablation except for LVEF (age [year]: 58 ± 14 vs. 50 ± 15, *p* = 0.901; LA diameter [mm]: 42 ± 9 vs. 38 ± 7mm, *p* = 0.299; LVEF [%]: 61 ± 5 vs. 58 ± 17, *p* = 0.040). In two patients with PAF, ablation targeting PLSVC resulted in acute termination of AF. As ablation within PLSVC was conducted in only nine patients, analysis of the correlation between ablation parameter setting and rate of successful isolation of PLSVC was not viable in our case series.

One patient with a history of PsAF for more than 10 years failed to restore SR despite of repeated electro cardioversion. Complete linear block was achieved in all LA rooflines and CTI lines. However, MI block failed in three patients after extensive ablation at the endocardial aspect and inside CS. Notably, ablation time at the MI region (both endocardial and epicardial aspects) was relatively long in patients with PLSVC (19 ± 5 min) (10). No procedure-related complications occurred in our cohort. Detailed baseline and procedural characteristics are demonstrated in Table 1.

**TABLE 1:** Baseline and procedural characteristics.

Patient number	Gender	Age *	Arrhythmia type	LA (mm)	LVEF (%)	Ablation strategy	Ectopies from PLSVC	PLSVC isolation	FU or time to recurrence (m)	Recurrence	Fluoroscopy time (min)	Procedure time (min)	PLSVC ablation time (min)	Power used in PLSVC ablation	MI ablation time (min)
#1	M	79	PsAF	39	55	2C3L + CFAE	No	No	50	No	5	180	/		18
#2	M	64	PsAF	49	65	2C3L + CS	No	No	40	Yes	5	180	/		20
#3	F	58	PAF	32	62	PVI	No	No	39	Yes	2	114	/		/
#4	M	59	PAF	45	58	PVI	No	No	38	Yes	0	123	/		/
#5	F	75	PAF	35	66	gap-closing for PVI	No	No	5	Yes	0	96	/		/
#6	F	51	PAF	36	62	PVI	No	No	32	Yes	3	120	/		/
#7	M	47	PsAF	43	53	2C3L + CFAE (failed MI block)	No	No	20	No	5	202	/		22
#8	F	70	PAF AVNRT	32	67	PVI	No	No	15	No	0	134	/		/
#9	F	30	PsAF	56	66	2C3L + CFAE + CS + PLSVC	/	/	/	Acute fail	5	198	28	25W	16
#10	F	58	PsAF	62	58	2C3L	No	No	6	No	9	150	/		14
#11	M	61	PAF	38	62	PVI + PLSVC-I	Yes	Yes	8	No	10	132	20	35W	/
#12	M	60	PAF	27	72	PVI + RSVC + PLSVC-I	Yes	Failed	7	No	12	154	32	25W	/
#13	M	47	PAF	44	66	3L + CFAE + PLSVC-I	Yes	Yes	6	Yes	8	148	26	30W	17
#14	M	57	PsAF	41	60	2C3L (MI block failed)	Yes	Yes	15	Yes	9	138	/		25
#15	M	26	PsAF	41	29	2C3L + PLSVC-I	Yes	Yes	3	Yes	0	180	29	40W	27
#16	M	47	PAF	40	57	PVI	No	No	4	Yes	3	108	/		/
#3 redo	/	/	PAF	/		LPV gap + PLSVC-I	Yes	Yes	20	No	7	132	19	35W	/
#14 redo	/	/	PAF	/		RPV gap + MI (block failed) + PLSVC-I	Yes	Yes	4	No	8	164	33	35W	15
#15 redo	/	/	AFL	/		PLSVC-I	Yes	Yes	3	No	11	220	16	35W	5
#16 redo	/	/	PAF	/		3L + CFAE + PLSVC-I	Yes	Yes	1	No	8	150	12	30W	13

AFL, atrial flutter; ASD, atrial septum defect; AVNRT, atrioventricular nodal reentrant tachycardia; CFAE, complex fractionated atrial electrogram; CS, coronary sinus; CTI, cavotricuspid isthmus; F, female; FU, follow up; LA, left atrium; LPV, left pulmonary vein; M, male; MI, mitral isthmus; PAF, paroxysmal atrial fibrillation; PLSVC-I, isolation of persistent left superior vena cava; PsAF, persistent atrial fibrillation; PVI, pulmonary vein isolation; PRV, right pulmonary vein; 2C3L, circumferential pulmonary vein isolation and linear ablation of the left atrial roof line, mitral isthmus line and cavotricuspid isthmus line (3L). *Age at the index ablation procedure for atrial fibrillation.

#### Follow-up and repeated ablation procedures

**FIGURE 2:**
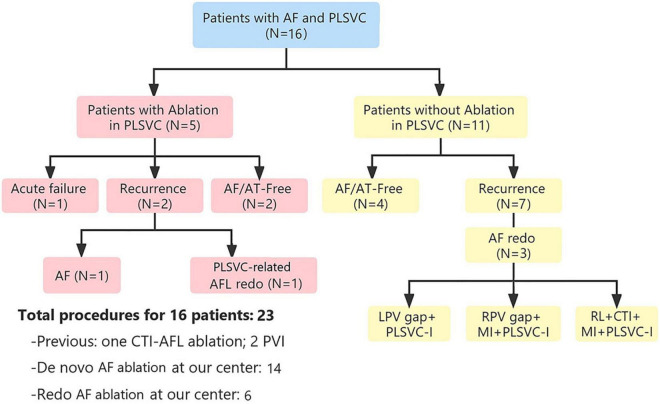
Flow diagram showing procedural and follow-up outcomes. AF, atrial fibrillation; AFL, atrial flutter; AT, atrial tachycardia; CTI, cavotricuspid isthmus; LPV, left pulmonary vein; PLSVC, persistent left superior vena cava; RPV, right pulmonary vein.

**FIGURE 3:**
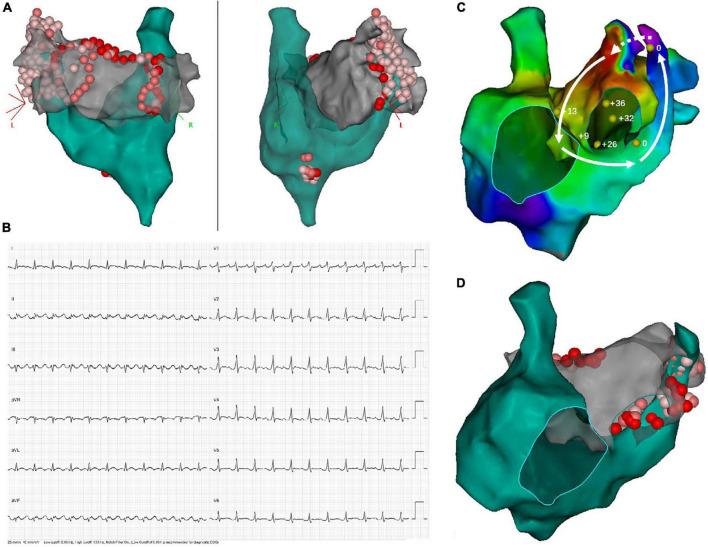
Demonstration of twice ablation procedure in a patient with persistent AF and PLSVC. **(A)** In the first ablation procedure, PVI, as well as linear ablation at the LA roofline, MI line, and CTI line was performed. Extensive ablation targeting LA-PLSVC and CS-PLSVC connection, as well as high-frequency signals inside PLSVC, resulted in PLSVC isolation. **(B)** ECG of recurrent atrial flutter (AFL). **(C)** Bi-atrial AFL involving a connection between PLSVC and left atrial appendage. Sites where entrainment mapping was conducted were indicated by yellow dots, with the numerical value of post-pacing interval minus tachycardia cycle length (PPI-TCL) labeled by white numbers. The dotted arrow indicated conduction through epicardial connections between the left atrial appendage (LAA) and PLSVC, while the solid lines with arrowhead indicated conduction pathway through LAA, the anterior wall of LA, interatrial septum, septal aspect of the right atrium, coronary sinus and PLSVC. **(D)** Repeated ablation targeting resumed LA-PLSVC connections, as well as touch-up ablation at the roofline, was conducted in the redo-procedure.

After a median follow-up period of 15 months (interquartile range [IQR], 6–38), only 6 (37.5%) patients remained AF/AT-free after a single ablation procedure, while 9/15 patients (excluding the one with acute failure) experienced recurrence, with a median ablation-to-recurrence time of 15 months (IQR, 4.5–38.5). Seven patients recurred as AF while two patients developed organized atrial tachyarrhythmia. A flow diagram summarizing the procedural and follow-up outcome was provided in Figure 2. One patient with sustained atrial flutter (AFL) underwent a redo-procedure three months after the index ablation. High-density activation mapping and entrainment mapping demonstrated a bi-atrial AFL with PLSVC constituting a part of the reentry circuit (Figure 3). Three patients undergoing redo-procedure for recurrent AF received successful PLSVC isolation and remained in SR since then. Detailed characteristics of each patient were listed in Table 1.

### Systematic review

#### Search results and quality assessment

**FIGURE 4:**
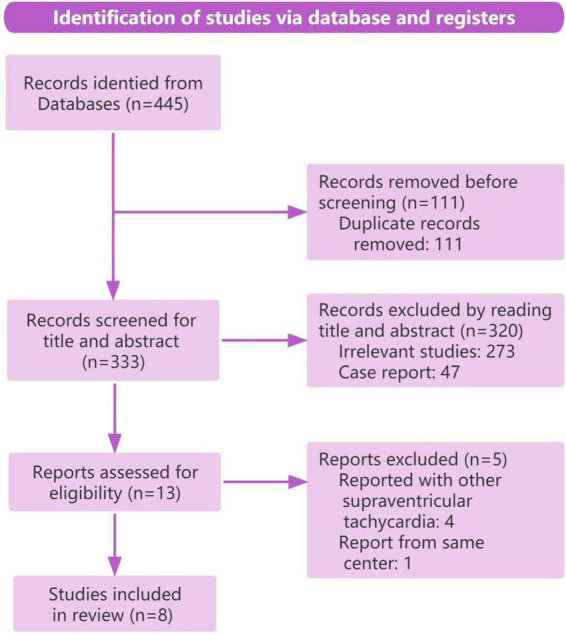
PRISMA flowchart.

Of 445 records retrieved by the searching strategy, 11 retrospective case series were eligible for the final analysis (4–6, 11–18), including three conference abstracts (16–18). The selection process is illustrated in Figure 4 (PRISMA). Quality appraisal of included studies is shown in Supplementary Table 2. According to the modified form of the Newcastle Ottawa Scale (9), a maximum of six criteria apply for the case series as shown in Supplementary Table 2. Six studies fulfilled all the six criteria (4, 5, 12–15), two studies fulfilled five criteria (6, 11), and one study fulfilled four criteria (16). Therefore, six articles were judged as good quality, two as sufficient quality, and one as intermediate quality (Supplementary Table 2). All authors agreed with this study classification.

#### Baseline characteristics

**FIGURE 5:**
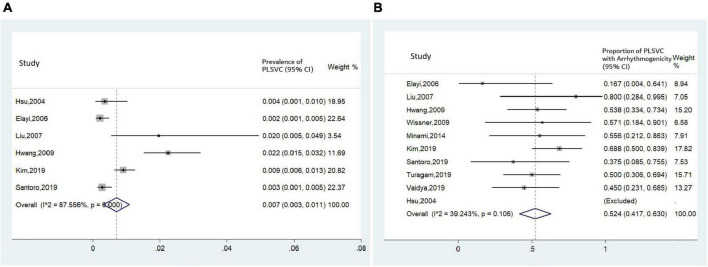
Forest plots assessing **(A)** prevalence of PLSVC in patients with AF receiving CA, and **(B)** proportion of arrhythmogenic PLSVC.

A total of 167 AF patients with PLSVC (58.4 ± 1.5 years, 69.5% male) were reported in the included studies. Noticeably, the average age of this population was much younger than the average age of AF diagnosis (75.8 ± 12.7 years) in the general population (19). The pooled prevalence of PLSVC in AF patients undergoing CA was 0.7% based on six case series (4, 6, 11–13, 17) (95% CI 0.3–1.1%, *I*^2^ = 87.6%, Figure 5A). Further baseline characteristics were shown in Table 1. After excluding patients on medical treatment and those receiving surgical procedures, 162 patients undergoing a total of 200 CA procedures were included in the procedural-related analysis, with 152 patients receiving radiofrequency ablation and 10 patients receiving cryoballoon ablation.

#### Procedural data

Mapping within PLSVC was performed in all studies. Two kinds of arrhythmogenic roles were brought forward: if ectopies from PLSVC, whether spontaneous or induced (e.g., by isoproterenol), could initiate a sustained AF, the PLSVC would be regarded as a trigger; while, if the shortest AF cycle was recorded in PLSVC during AF onset, then the PLSVC would be deemed as a driver, or perpetuator, of AF (6). Pooled analysis revealed that the proportion of ‘arrhythmogenic PLSVC’ was estimated to be 52.4%, with a moderate heterogeneity among studies (*I*^2^ = 39.2%) (Figure 5B).

The summarization of details of ablation procedures were displayed in Table 2.

**TABLE 2:** Baseline characteristics of the enrolled studies.

Study	PLSVC/ Total AF (n/N)	Procedures	Age (y)	Male (n/N)	PAF (n/N)	AF history (m)	LA (mm)	CHD	Prior ablation	SVC abnormality	Concomitant -arrhythmia
Hsu et al. (11)	5 (3/851) *	5	46 ± 11	4/5	4/5	146 ± 77	NR	1 ASD 1 PAPVD	1 typical AFL; 1 incisional AFL	NR	no
Elayi et al. (4)	6/2820	6	50 ± 6.4	4/6	4/6	NR	41 ± 4	None	9 PVI in 4 pts	none	1 SSS
Liu et al. (12)	4/204	9	50 ± 12	0/4	4/4	74 ± 32	50 ± 12	NR	None	3 RSVC atresia	1 tricuspid AFL
Hwang et al. (17)	29/1293	26	55 ± 13	22/29	NR	NR	NR	NR	NR	8/29 pts had a small left subclavian vein connection to RSVC	4 AVNRT 2 septal AT 4 RA-AFL
Wissner et al. (5)	7	14	57 ± 8	4/7	2/7	NR	43 ± 6	1 ASD 1 VSD	None	NR	1AVNRT
Minami et al. (16)	9	9	53 ± 10	27/36	9/9	NR	NR	NR	None	NR	NR
Kim et al. (6)	36/3967	46	62 ± 12	6/8	19/36	64 ± 28	NR	4 ASD 3 PAPVD 2 VSD 1 RPV atresia	None	2 RSVC atresia; 31 dual SVCs: With anastomosis: 15 No anastomosis: 16 2 PLSVC draining into LA;	9 RA-AFL 5 AVNRT 2 septal AT&LA-AFL junctional AT 1 AVNDP, 1 SSS
Santoro et al. (13)	8/2876	10	65 ± 7	22/28	2/8	NR	44 ± 4	NR	None	2 RSVC atresia	3 PM for unknown indications
Turagam et al. (14)	28	28	61 ± 8	8/15	17/28	60 ± 33	44 ± 8	NR	11 PVI, 2 PLSVC ablation	3 PLSVC draining into LA	NR
Vaidya et al. (18)	20	20	56 ± 12	13/20	NR	NR	NR	11 pts	NR	NR	NR
Kantenwein et al. (15)	15	27	65 ± 15	6/9	9/15	NR	NR	2 PFO	7 AF ablation in 3 pts	3 CS ostium atresia, 3 lacking RSVC	NR

*Prevalence data is available only at 1 center. AFL, atrial flutter; ASD, atrial septal defect; AT, atrial tachycardia; AVNDP, atrioventricular nodal dual path; AVNRT, atrioventricular nodal reentry tachycardia; CHD, congenital heart disease; CS, coronary sinus; NR, not reported; SSS, sick sinus syndrome; PAF, paroxysmal atrial fibrillation; PAPVD, partial anomalous pulmonary venous drainage; PFO, patent foramen ovale; PM, pacemaker; PVI, pulmonary vein isolation; RA, right atrium; RPV, right pulmonary vein; RSVC, right superior vena cava; SVC, superior vena cava; VSD, ventricular septal defect.

**FIGURE 6:**
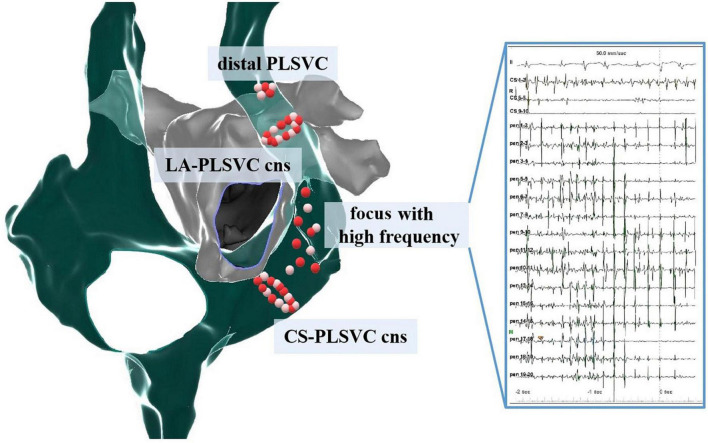
Distribution pattern of ablation sites within PLSVC. Cns, connections; LA, left atrium; CS, coronary sinus.

Ablation strategies varied among different studies (Table 3). To interrupt the LA-PLSVC connections, ablation at the mid-portion of PLSVC was performed, while extensive ablation at distal PLSVC as well as LA endocardium was occasionally necessitated (14). CS-PLSVC connections would be eliminated at the proximal PLSVC. In some studies, high frequency signals in PLSVC were targeted (5, 12, 14). A pattern diagram showing the distribution of ablation lesions in PLSVC is developed based on studies giving a specific description of the ablation sites (Figure 6).

**TABLE 3:** Procedural characteristics.

Study	Arrhythmogenic PLSVC	Fluoroscopy time (min)	Procedure time (min)	Ablation time (min)	Energy	Mapping system	Catheters and parameters of PLSVC ablation
Hsu et al. (11)	5/5	NR	NR	CS-PLSVC: 11 ± 3 LA-PLSVC: 9 ± 3	RF	Lasso CARTO	4-mm conventional/irrigated catheters; 50°C, 25 W
Elayi et al. (4)	1/6	NR	NR	10.25 ± 1.6 in PLSVC	RF	Lasso	8-mm catheter, 50°C, 50 W
Liu et al. (12)	4/5	NR	NR	PLSVC: index-procedures: 16 ± 12 redo-procedures: 9 ± 6	RF	Lasso CARTO	5 mm or 3.5-mm irrigated catheters; 65°C, 30 W, 30 mL/min
Hwang et al. (17)	14/26	NR	NR	NR	RF	3D EAM	NR
Wissner et al. (5)	4/7	NR	NR	NR	5 RF, 2 CB	CARTO	RF: irrigated catheter, 43°C, 20 W, 17 mL/min CB: 28m m balloon, 300 s, –80°C
Minami et al. (16)	5/9	NR	NR	NR	NR	NR	NR
Kim et al. (6)	22/32	37.5 ± 10.7	224 ± 32	Total: 49.7 ± 26.6, PLSVC: 32.0 ± 13.9	RF	NR	22.7 ± 1.3W
Santoro et al. (13)	3/8	32 ± 18	120 ± 22	PLSVC-i: 61s/125s; Freeze cycle duration: 180s/180s/300s	CB	NR	28 mm balloon, 180–300 s, –60°C
Turagam et al. (14)	14/28	30.6 ± 9.8	253 ± 35	NR	RF	CARTO Ensite Rhythmia	irrigated catheters 43°C, 15–20 W, 17 ml/min
Vaidya et al. (18)	9/20	NR	NR	NR	NR	NR	NR
Kantenwein et al. (15)	NR	11.5 ± 6.0	175 ± 48	Total: 48.9 ± 16.1	RF	EnSite CARTO Rhythmia	20–30W

CB, cryoballoon; RF, radiofrequency; NR, not reported.

In the 162 patients undergoing CA, a total of 121 (74.7%) patients receiving ablation in PLSVC, with 55 patients reported to achieve PLSVC isolation (4, 11–14, 16, 18). Five patients failed to have complete PLSVC isolation, and two patients received only focal ablation (18), while in the remaining 66 patients no clear clarification was available on whether PLSVC isolation was achieved (5, 6, 15, 17).

#### Safety outcomes

A total of 15 complications (7.5%) were reported in six studies (5, 6, 13–15, 18). Major complications included four cases of cardiac tamponades (2%), three cases of cardiac effusion (1.5%), three cases of phrenic nerve injury (1.5%) (one left phrenic nerve [LPN] and two right phrenic nerve [RPN]), and one ischemic stroke.

#### Follow-up and efficacy outcomes

The mean follow-up (FU) duration of seven studies reporting with mean and standard deviations was 23.4 months (95% CI: 15.2–31.7). Three case series reported outcomes after a fixed FU period [1 year (14, 18) and 332 days (13)], while the FU period was not clarified in one study (15) (Table 4).

**TABLE 4:** Ablation strategy and procedure outcome.

Study	Ablation strategy	Ablation targets in PLSVC	Rate of PLSVC ablation (PLSVC-a) and isolation (PLSVC-i)	Complications	Follow-up		Recurrence
					Period (m)	Time points and methods	
Hsu et al. (11)	PVI + PLSVC	CS-PLSVC Cns LA-PLSVC Cns	PLSVC-a in all 5 pts, PLSVC-i in 4/5 pts	None	15 ± 10	At regular intervals (unspecified); 12-lead and ambulatory ECG	1 AF (failed PLSVC-i), 1 LA flutter
Elayi et al. (4)	PVI + PLSVC	CS-PLSVC Cns LA-PLSVC Cns	PLSVC-i in all 6 pts	None	13 ± 7	rhythm transmitters; 12-lead ECG, and 48 h Holter monitoring at 3, 6, and 12 months after ablation;	none
Liu et al. (12)	PVI + PLSVC	PLSVC potentials	PLSVC-i in all 4 pts	None	18 ± 7	Clinic visit with symptom recurrence; 24 h Holter monitor at 6–9 months after ablation procedure	3/4 pts in SR after a median of 2 (2–3) procedures
Hwang et al. (17)	NR	LA-PLSVC Cns	PLSVC-a in all 26 pts, PLSVC-i: NR	NR	15.6 ± 5.5	Not specified	18/26 pts in SR
Wissner et al. (5)	PVI, conditional CFAE, conditional PLSVC	High-frequency signals at mid-to proximal PLSVC	PLSVC-a in 3/7 pts, 7/14 procedures; PLSVC-i: NR	1 LPN injury, 1 cardiac tamponade, 1 ischemic stroke	23.8 ± 11.6	12-lead ECG and 24h Holter monitoring 1, 3, and 6 m after the procedure and at 6m intervals; event recorder	5/7 pts in SR after a median of 2 (1–4) procedures
Minami et al. (16)	PVI, conditional PLSVC	NR	PLSVC-a PLSVC-i in 5/9 pts	NR	16.0 ± 9.8	Not specified	7/9 pts in SR
Kim et al. (6)	PAF: PVI + trigger + conditional PLSVC; PsAF: PVI + conditional linear/CFAE/ PLSVC	Circumferential ablation at mid-PLSVC, LA-PLSVC Cns, High-frequency signals	PLSVC-a in 26/32 pts; PLSVC-i: NR	3 cardiac tamponades	74.0 ± 40.2	12-lead ECG at 1, 3, and 6 m after the procedure and at 6m intervals thereafter; event recorder	22/32 pts in SR
Santoro et al. (13)	PVI; conditional PLSVC	NR	PLSVC-a in 3/8 pts, PLSVC-i in 2/8 pts	2 RPN injury	332 days	12-lead ECG and 24h Holter monitoring 1, 3, and 6 m after the procedure and at 6m intervals; CIED interrogation; telephone interviews in cases of recurrence symptoms	63.5% pts in SR
Turagam et al. (14)	PVI ± focal/lines; CFAE in all PsAF	Mid-PLSVC; High-frequency signals; Proximal CS; Endo-LA at operators’ discretion	PLSVC-a in 28 pts, PLSVC-i in 27 pts	1 minor pericardial effusion; 3 groin hematomas	1 year	12-lead ECG and 24h Holter monitoring 1, 3, 6, and 12 m after the procedure	75% pts in SR
Vaidya et al. (18)	NR	NR	PLSVC-a in 9/20 pts, PLSVC-i in 7/20 pts	2 pericardial effusions	1 year	Not specified	65% pts in SR
Kantenwein et al. (15)	PVI ± CFAE/line/ PLSVC	NR	PLSVC-a in 6/15 pts, 13/27 procedures, PLSVC-i: NR	1 atrial septum dissection	NR	Not specified	2nd ablation needed in 9 pts 3rd ablation needed in 3 pts

Abbreviations same as Table 1. CIED, cardiac implanted electronic device; LPN, left phrenic nerve; NR, not-reported; RPN, right phrenic nerve; SR, sinus rhythm.

In nine studies with elaborated records on the ablation times for each patient, 86/124 (69.3%) patients underwent a single ablation procedure, while 30/124 (24.1%) patients received a redo-procedure. A third procedure was required in six patients and a fourth in two patients.

**FIGURE 7:**
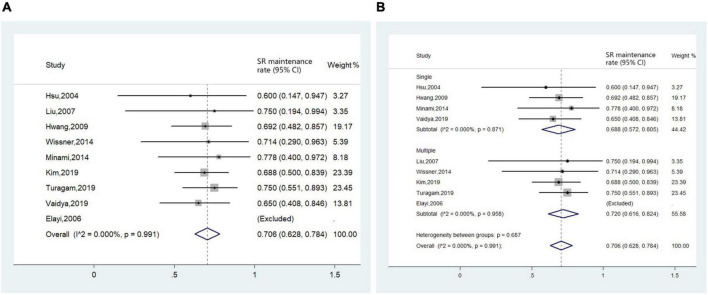
Forest plot of the included studies for the efficacy endpoint of **(A)** long-term sinus rhythm (SR) maintenance rate in all patients and **(B)** subgroup analysis of long-term sinus rhythm maintenance rate based on times of ablation procedures (single procedure and multiple procedures).

The efficacy endpoint evaluated by AF/AT-free rate was available in 10 studies (4–6, 11–14, 16–18). We assessed the long-term outcome of CA for AF in nine studies with a FU period equal to or longer than 1 year. Pooled analysis revealed that after a median follow-up period of 15.6 months (IQR 12.0–74.0 months), the long-term AF/AT-free rate was 70.6% (95% CI 62.8–78.4%, *I*^2^ = 0.0%, Figure 7A). Subgroup analysis conducted in studies reporting AF/AT-free rate after a single procedure and studies including part of patients receiving multiple procedures yielded a similar result (Figure 7B).

Eight studies documented a total of 55 redo procedures (4–6, 11–15). Intervention targeting PLSVC was necessitated in most redo-procedures (38/55, 69.1%), with either re-isolation of recovered activities of PLSVC, or *de novo* isolation for a previously omitted arrhythmogenic PLSVC.

#### Publication bias

**FIGURE 8:**
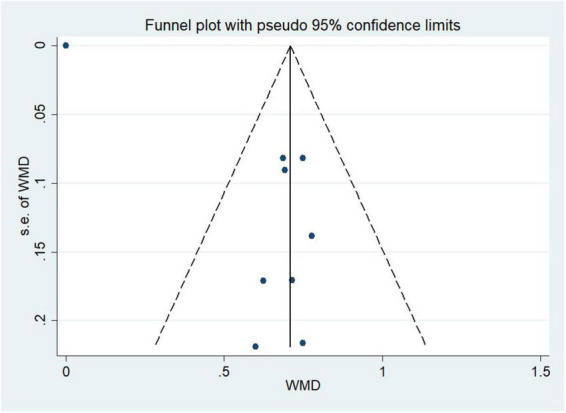
Publication Bias-Funnel plot for studies evaluating sinus rhythm maintenance rate.

Funnel plots combined with Egger’s test were created for the examination of publication bias. The *p*-value in Egger’s test was 0.573, suggesting that there was no proof of publication bias, as shown in Figure 8.

## Discussion

To our knowledge, this is the first systematic review on CA for AF in patients with PLSVC. The major findings of the cohort study along with the systematic review include:

1) The prevalence of PLSVC in AF patients was estimated to be 0.7%. Over half of PLSVCs were confirmed to play critical arrhythmogenic roles in the initiation or maintenance of AF;
2) Ablation in PLSVC was necessitated in most AF patients with PLSVC, mostly targeting LA-PLSVC and CS-PLSVC connections, as well as sites with high-frequency signals;
3) CA for AF in patients with PLSVC was generally safe and efficacious. The incidence of procedural complications (7.5%) and AF/AT-free rate during long-term following-up (over 70%) was comparable to that of general AF population (20);
4) Repeated ablation was common in AF patients with PLSVC (up to 30% estimated from available data). Recovery of previous isolated PLSVC or omitted arrhythmogenic PLSVC accounted for the most common causes of arrhythmia recurrence.

### Prevalence of persistent left superior vena cava in atrial fibrillation patients

To date, there hasn’t been an accurate report on the prevalence of PLSVC in AF patients. We for the first time made an estimation based on current studies. However, it should be noticed that prominent heterogeneity existed among included studies. In addition, the true prevalence of PLSVC in AF patients might be higher than this, considering that some less obvious PLSVC might be missed out.

As some thin PLSVCs are inconspicuous on routine transthoracic echocardiography, intraprocedural observation of PLSVC is also of great significance. Some abnormal signs may hint at the existence of PLSVC, including (1) abnormally enlarged CS observed during catheter positioning; (2) AF sustaining despite isolated pulmonary veins, (3) AF onset at a young age without clear causes, especially in patients with other cardiac development anomalies [i.e., atrioventricular septal defects, conotruncal malformations, and left-sided defects (21)]. Additionally, with the increasing use of ethanol infusion into the vein of Marshall during ablation for persistent AF, PLSVC might be discovered during the venogram of the CS. In these cases, ICE can provide much useful anatomical information.

### Anatomical and electrophysiological characteristics of persistent left superior vena cava

The PLSVC courses between the LA appendage (LAA) and the left superior pulmonary vein before draining into the right atrium via an enlarged CS. Occasionally, drainage into LA can also occur. In most cases, bilateral SVCs coexist with or without an anastomosis through an innominate vein, while in some rarer cases, PLSVC presents with a concomitant absence of the RSVC (6).

Based on current evidence, PLSVC can participate in the genesis and maintenance of AF through three distinct electrophysiological properties:

(1) Triggering activity: tissue with pacemaker activities exists bilaterally near the sinus horns and common cardinal veins during the embryological period, which can be preserved in the undegenerated PLSVC (22), rendering its autorhythmicity. Measures like high-dose isoproterenol infusion (20–30 mg/min for 10–15 min) (23) can help to adequately expose the triggering focus including those from PLSVC (4, 6, 11, 17).

(2) Perpetuator of AF: the complex muscular structure in PLSVC endues it with the potential to be a perpetuator during AF persistency. In some studies, ablation targeting sites with high-frequency signals or CFAE within PLSVC was performed (5, 24).

(3) Connections with LA and CS: as revealed by histological studies, extensive muscular connections exist between LA and PLSVC (25), thus, ectopies from PLSVC can propagate through these connections and subsequently initiate episodes of AF. In addition, LA-PLSVC connections can also serve as the critical isthmus of the LA flutter (18, 26, 27) and increase difficulties in achieving a complete MI block (28). Thus, a thorough elimination of these connections is of critical importance. A meticulous mapping for the earliest activation site, combined with pacing at low output and observing the capture of adjacent structures can locate these connections. Although Hsu et al. (11) reported an average of 1.6 ± 0.5 LA-PLSVC connections and 4.1 ± 2.3 CS-PLSVC connections per patient, extensive ablation at the middle and proximal portion of PLSVC, or even at distal PLSVC and endocardium of LA was required to disrupt these connections.

### Importance of persistent left superior vena cava isolation in atrial fibrillation ablation

Based on current evidence, PLSVC acted as an initiator or perpetrator of AF in more than half cases and was a common cause of arrhythmia recurrence. Therefore, achieving PLSVC isolation could be considered routine practice in highly experienced centers with adequate safety guarantees. At least, PLSVC isolation should be performed if it is found to be arrhythmogenic.

Several phenomena can be observed after the achievement of PLSVC isolation: (1) alteration in the activation sequence of PLSVC during LA pacing, which corroborates a disconnection between LA and PLSVC (6, 29); (2) failure to capture the LA during pacing within PLSVC and vice versa (6, 11); (3) loss or dissociation of the local venous potential from the PLSVC (5, 12). When assessing the isolation of PLSVC using the third criteria, one should be aware that the presence of a dissociated potential within PLSVC during AF/AT onset can only testify an entrance block but not an exit block, since spontaneous activity within PLSVC is likely to be overridden and therefore could not manifest any exit conduction (29).

### Potential challenges of ablation within persistent left superior vena cava

Although in the study by Wissner et al. (5) the incidence of complications was impressively high (3/7) in patients undergoing PLSVC ablation, the overall complication risk was acceptable considering the pooling data. The most specific risk carried with ablation inside PLSVC is left phrenic nerve (LPN) injury. As LPN descends along the anterolateral aspect of PLSVC, injury of LPN should be watched out for when ablating at an anterolateral site, especially in the mid-to-distal portion of PLSVC (30, 31). Pacing at the maximal output through the ablation catheter should be performed to confirm that the ablation point is away from LPN every time before energy delivery (6, 13). The occurrence of cardiac tamponade was also relatively high (2%) in current studies. Thus, judicious control of contact force and ablation power during RF ablation is required during ablation within delicate structures like CS and PLSVC.

In addition, to achieve isolation of PLSVC, ablation targeting the CS-PLSVC connection is indicated. According to histologic examinations by Kim et al. (32), muscular connections between Ligament of Marshall (vestige of PLSVC) and CS exist around proximal CS near the origin of the Vein of Marshall, which, according to another study, locates 29.1 ± 9.6 mm from the CS ostium (25). Although this distance seems enough in anatomically normal heart, in patients with PLSVC, the difficulties and risks of ablation in the vicinity of CS ostium are much higher due to an enlarged CS, which results in a distorted Triangle of Koch and abnormalities of the location of atrioventricular (AV) conduction system. His potential could be recorded at the upper border of CS ostium (as illustrated in the Central Illustration), and ablation in the vicinity of the enlarged CS ostium could induce persistent accelerated junctional rhythm (33). Therefore, the operator should remain cognizant of the possibility of damaging AV conduction ability during ablation of the CS-PLSVC connection. In addition, altered anatomical relationship of structures surrounding CS ostium also cause much challenges to catheter stability (34).

### Further research perspectives

Eliminating the multiple connections between PLSVC and LA as well as CS in a point-by-point fashion by RF ablation may be time-consuming and tends to result in incomplete isolation. To this end, a ‘one-shot’ ablation tool might be a better solution to this challenging issue. Cryoballoon ablation has been reported in several studies (5, 13, 35, 36). Other types of ‘one-shot’ ablation tools, like the Pulmonary Vein Ablation Catheter (PVAC, Medtronic, Minneapolis, MN, USA) (37) and a decapolar irrigated circular catheter (nMARQ, Biosense Webster, Diamond Bar, CA, USA) (38) has also been employed in the ablation in PLSVC. Recently, there was a pilot report on the usage of pulsed field ablation (PFA) in the isolation of PLSVC (39). With the advance in ablation techniques and tools, more choices are available for PLSVC isolation and should be tested in clinical practice.

### Limitations

Several limitations in the present systematic review need to be acknowledged. In the case series, the proportion of PLSVC showing spontaneous triggering activities in our case series was lower than previously reported, which may account for the higher recurrence rate in our center. In fact, the missed triggers in PLSVC may play a critical role in the development of AF in this specific population, as reflected by the necessity of PLSVC ablation in all four redo cases. Drug challenges using isoproterenol or adenosine may be more effective to reach the threshold of arrhythmia inducibility for triggers or automatic mechanism than burst atrial pacing (40). In addition, as we do not routinely evaluate PLSVC by cardiac CT or magnetic resonance, we could not establish a relationship between the anatomical characteristics of PLSVC and its arrhythmogenicity. Our case series (as a negative example) and previous studies all highlight the important to seek extra-PV triggers intentionally in patients with PLSVC.

In addition, the quality of the systematic review is limited by the nature of small case series of included studies. Great heterogeneity existed among available studies, including ablation techniques and tools, mapping systems, ablation strategies, parameter settings as well as variable follow-up and outcome measurements, and the scale of the included case series are rather small. With these limitations, the reliability and robustness of the pooled analysis might be hampered.

## Conclusion

Although the prevalence of PLSVC is low in AF patients, it is common to play an arrhythmogenic role in the initiation or maintenance of AF. CA of AF in patients with PLSVC usually involves intervention targeting LA-PLSVC and CS-PLSVC connections, as well as focal with high-frequency signals. Overall, CA can result in an acceptable rate of atrial arrhythmia freedom with relatively low risk of complications. However, current evidences are derived from small non-controlled cohort studies. Future well-designed randomized controlled trials or large-scale registries are still needed to explore the optimal interventional strategy for AF in patients with PLSVC.

## Central illustration

Catheter ablation of atrial fibrillation in patients with persistent left superior vena cava.

## Data availability statement

The raw data supporting the conclusions of this article will be made available by the authors, without undue reservation.

## Ethics statement

The studies involving human participants were reviewed and approved by Ethnic Institute of Beijing Anzhen Hospital. The patients/participants provided their written informed consent to participate in this study.

## Author contributions

SL, CM, and JD: contributing to the conception and design. MG and YB: drafting the manuscript. LH, JZ, CL, NL, XL, SZ, XG, WW, and XZ: data collection, analysis, and interpretation. DL, CS, and RT: revising the manuscript. All authors contributed to the article and approved the submitted version.
