# Supplementary material for: Catheter ablation for atrial fibrillation in patients with persistent left superior vena cava: Case series and systematic review
Source: Front Cardiovasc Med. 2022 Oct 17;9:1015540. doi: 10.3389/fcvm.2022.1015540 (PMC9632661; doi:10.3389/fcvm.2022.1015540)
Supplement: Supplementary file 1 [file Data_Sheet_1.docx]

**Systematic Review Process**

A detailed protocol was developed and was registered in the international prospective register for systematic reviews (PROSPERO) before search execution (CRD 42022341242). Principles of Preferred Reporting Items for Systematic Reviews and Meta-Analysis guidelines for reporting (PRISMA)(1) and the Cochrane Handbook for Systematic Review of Interventions(2) were strictly followed. Inclusion was restricted to English literatures. Reviews, editorials, letters, meta-analysis, case reports were excluded. Case series required a minimum of three patients to be considered eligible. Identified records were imported into Endnote (20.0, Clarivate Analytics, Philadelphia, PA) and duplicate records were removed.

**Supplementary Table 1** Complete searching strategies and results.

| Date of search: 2 July, 2022 | | |
| --- | --- | --- |
| PubMed/Medline | | |
| #1 | "persistent left superior vena cava"[MeSH Terms] OR "left sided superior vena cava"[ti/ab] OR "PLSVC"[ti/ab] OR "persistent LSVC"[ti/ab] OR "bilateral SVC"[ti/ab] | 541 |
| #2 | "atrial fibrillation"[MeSH Terms] OR "atrial fibrillations"[ti/ab] OR "fibrillation atrial"[ti/ab] OR "persistent atrial fibrillation"[ti/ab] OR "paroxysmal atrial fibrillation"[ti/ab] | 68,161 |
| 3 | (#1) AND (#2) | 15 |
| Embase | | |
| #1 | 'persistent left superior vena cava'/exp | 1,858 |
| #2 | 'left superior caval vein':ti,ab,kw OR 'left superior vena cava':ti,ab,kw OR 'persistent left superior cava vein':ti,ab,kw OR 'persistent left superior caval vein':ti,ab,kw OR 'persistent left superior vena cava':ti,ab,kw | 2,496 |
| #3 | #1 OR #2 | 3,010 |
| #4 | 'atrial fibrillation'/exp | 192,526 |
| #5 | #3 AND #4 | 230 |
| Web of Science from 1900 | | |
| #1 | TS=(left* NEAR/2 vena cava) OR TS=(left* NEAR/2 cava*) | 3634 |
| #2 | TS=(atrial fibrillation)) OR TS=(atria* NEAR/3 (fibrillat* or flutter* or ar?hythm* or tachycard* or tachyar?h*) | 174,575 |
| #3 | (#1) AND (#2) | 200 |

**Supplementary Table 2** Quality appraisal of included studies by a modified form of the Newcastle Ottawa Scale(3).

| **Study** | **Points and reasons for point loss assess by the tool Murad 2017** | **Overall judgment** |
| --- | --- | --- |
| Hsu, 2004 | 5/6†, unclear selection approach led to -1 in the selection domain | Sufficient quality  (5 patients) |
| Elayi, 2006 | 6/6† | Good quality  (6 patients) |
| Liu, 2007 | 6/6† | Good quality  (4 patients) |
| Wissner, 2010 | 6/6† | Good quality  (7 patients) |
| Minami, 2014 | 4/6†, unclear outcome ascertainment, case not described with sufficient details | Intermediate quality  (9 patients) |
| Kim, 2019 | 5/6†, no report on procedure-related complications | Sufficient quality  (32 patients) |
| Santoro, 2019 | 6/6† | Good quality  (8 patients) |
| Turagam, 2019 | 6/6† | Good quality (28 patients) |
| Kantenwein, 2022 | 6/6† | Good quality  (15 patients) |
| †: Question 5 and 6 of the score are not applicable in this study, therefore 6 is the maximum score instead of 8 | | |

**Reference:**

1. Page MJ, McKenzie JE, Bossuyt PM, Boutron I, Hoffmann TC, Mulrow CD, et al. The PRISMA 2020 statement: an updated guideline for reporting systematic reviews. BMJ 2021;372:n71.

2. Higgins JPT TJ, Chandler J, Cumpston M, Li T, Page MJ, Welch VA. Cochrane Handbook for Systematic Reviews of Interventions version 6.3 (updated February 2022). Cochrane, 2022.

3. Murad MH, Sultan S, Haffar S, Bazerbachi F. Methodological quality and synthesis of case series and case reports. BMJ Evid Based Med 2018;23:60-63.
